# HLA Genetic Diversity and Chronic Hepatitis B Virus Infection: Effect of Heterozygosity Advantage

**DOI:** 10.3390/medsci12030044

**Published:** 2024-08-29

**Authors:** Adriana Tălăngescu, Maria Tizu, Bogdan Calenic, Dan Florin Mihăilescu, Alexandra Elena Constantinescu, Ileana Constantinescu

**Affiliations:** 1 Immunology and Transplant Immunology, “Carol Davila” University of Medicine and Pharmacy, 020021 Bucharest, Romania; adriana.oprea@drd.umfcd.ro (A.T.); bcalenic@yahoo.co.uk (B.C.); alexandra.constantinescu08@gmail.com (A.E.C.); ileana.constantinescu@imunogenetica.ro (I.C.); 2 Centre of Immunogenetics and Virology, Fundeni Clinical Institute, 258 Fundeni Avenue, 022328 Bucharest, Romania; 3 Department of Anatomy, Animal Physiology and Biophysics, Faculty of Biology, University of Bucharest, Splaiul Independentei Street, No. 91–95, 050095 Bucharest, Romania; d.f.mihailescu@gmail.com; 4 “Emil Palade” Centre of Excellence for Young People in Scientific Research (EP-CEYR), 3 Ilfov Street, Sector 5, 050045 Bucharest, Romania; 5 Academy of Romanian Scientists (AOSR), 3 Ilfov Street, Sector 5, 050045 Bucharest, Romania

**Keywords:** HBV infection, HLA diversity, heterozygote advantage

## Abstract

This research aims to determine whether HLA heterozygosity confers a protective effect against hepatitis B virus infection by analyzing the relationship between HLA diversity and the risk of hepatitis B virus (HBV) infection. A total of 327 hepatitis B patients were selected and categorized based on their clinical status: 284 patients with chronic HBV infection and 43 patients with HBV-related liver cirrhosis (LC). The control group included 304 healthy individuals. HLA genotyping for 11 loci, including HLA class I and class II, was conducted using next-generation sequencing. The results of this study indicate a statistically significant negative correlation between HLA class II heterozygosity and the risk of HBV infection. Specifically, heterozygosity in HLA-DQB1 (OR = 0.49, 95% CI = 0.31–0.76, *p* = 0.01277) and HLA-DRB1 (OR = 0.42, 95% CI = 0.24–0.77, *p* = 0.01855) were significantly associated with protection. Subgroup analysis was conducted to explore the effect of HLA diversity among pathological subtypes (chronic hepatitis B and control group, liver cirrhosis and control group). For liver cirrhosis, compared with the control group, a decreased risk of LC was possibly associated with the heterozygosity of HLA class I locus B (OR = 0.24, 95% CI = 0.09–0.65, *p* = 0.0591), but this hypothesis was not confirmed by other studies. The diversity of HLA, measured by HLA heterozygosity, was associated with a protective effect against HBV infection.

## 1. Introduction

Viral hepatitis B (HBV) is a significant problem for the public health system, not only in Romania but across the globe. Recent World Health Organization data indicate that around 257 million people worldwide are carriers of HBV. Individuals suffering from chronic HBV infection face an increased risk of complications, such as developing cirrhosis or hepatocellular carcinoma (HCC), with the degree of risk influenced by both the characteristics of the host and the virus [1]. The rate of chronic hepatitis B infection in Romania is notably high, presenting a significant public health concern. In fact, 4.4% of the population tests positive for the HBs antigen, indicating active hepatitis B infection, while 27% show anti-HBc antibodies, reflecting either past or ongoing infection. To date, the precise mechanisms underlying hepatitis B virus pathogenesis remain unclear. The course of the disease is influenced by a multitude of factors, including viral characteristics, environmental exposures, and host genetic factors. Understanding how these elements interact is crucial for developing effective strategies to manage and treat HBV infection.

The major histocompatibility complex, responsible for the genes that encode the information for the human leukocyte antigens (HLAs), is the most important for the well-being of the immune system. HLA class I proteins have a valuable part in presenting endogenous peptides to CD8+ T cells, leading to the cytotoxic death of the infected cells. Meanwhile, HLA class II peptides, found only on certain cells specialized in antigen presentation, trigger the activation of T helper lymphocytes by presenting exogenous peptides to these immune cells. These roles make HLAs vital for immune recognition and regulation and for distinguishing between self and non-self. To discover in detail the correlations between the HLA genes and how they influence the severity of the disease, the susceptibility, and especially the response we expect after the treatment of patients with certain HLA alleles, many studies have been performed. These associations have been extensively studied for various pathologies, populations, and geographical regions. Many studies have analyzed the impact of HLA genes on the evolution of HBV for different populations, and the results vary greatly from one study to another. In relation to hepatitis B infection, HLA genotypes have been proven to influence and modulate the outcome of the infection. Thus, certain alleles, such as class I (A*03:01) and class II (DRB1*13:01 and DRB1*13:02), have been associated with increased viral clearance, offering a protective effect against HBV infection [2,3]. On the other hand, the alleles on the DQ loci (DQB1*03:01, but also DQA1*05:01), and especially the DRB1*11:02 allele, have been associated with an increased susceptibility to developing HBV. Several studies in Asia [4,5] have shown that Chinese patients with HLA B*48 in association with DRB1*48 have a higher risk of hepatitis, as do Japanese individuals with certain gene variations of HLA-DP and HLA-DQ [6]. In contrast, other studies [7,8] have observed associations between viral persistence in patients with HLA B*08-B*44 genotypes in Caucasian patients, while for the same population, HLA B*58 offers protection alongside other HLA class II alleles, DRB1*13:01 and DRB1*13:02.

Research has also investigated other populations with various findings: Turkish patients with chronic HBV are more likely to express the HLA B*35 allele [9], Iranian HBV carriers frequently express the B*52 allele, compared to healthy individuals, and in Africa, the Gambian population seems to have an increased protection, due to the presence of DRB1*13:02 [10].

In a recent study conducted by our team, we identified a significant association between specific HLA alleles, including DQ alleles (DQB1*06:03:01, DQB1*06:02:01, and DQA1*01:03:01), along with DR alleles (DRB1*13:01:01, DRB1*15:01:01, and DRB5*01:01:01) and a reduced risk of hepatitis B virus infection in the Romanian population [11].

In evolutionary biology, there is a hypothesis that individuals with different alleles of HLA genes (heterozygotes) account for the significant polymorphism observed at various MHC loci and the long-term persistence of this genetic diversity. Heterozygous HLA genotypes enable a broader presentation of antigens for T lymphocyte recognition, compared to homozygotes with only one type of MHC molecule. This idea, presented to the world by a talented team conducted by Doherty and Zinkernagel, is congruent with the idea that heterozygosity contributes to increased resistance to infectious diseases by enhancing the immune system’s response to pathogens [12]. The heterozygote advantage has been analyzed in many diseases, such as colorectal cancer [13]; patients with late-stage HIV-1-related conditions [14]; and patients with ulcerative colitis [15].

The diversity of HLA, assessed through HLA heterozygosity and HLA evolutionary divergence (HED), has been linked to the susceptibility of various diseases, especially those with infectious or autoimmune etiologies. However, to the best of our knowledge, the heterozygote advantage in hepatitis B virus infection has not been previously investigated in the Romanian population.

The present study specifically aims to evaluate the heterozygote advantage hypothesis in hepatitis B infections by examining the depth of the relationship between the diversity of HLA genes and the severity of HBV infection.

## 2. Materials and Methods

### 2.1. Patients and Controls

Our study was elaborated and conducted at the Department of Gastroenterology and Hepatology, Fundeni Clinical Institute, between June 2022 and January 2024. In total, 327 patients with hepatitis B were consecutively selected and divided according to their clinical status as follows: 284 patients with chronic HBV infection and 43 patients with HBV-related liver cirrhosis (LC). Family members were not included in the study. The control group consisted of 304 uninfected healthy controls with/without HBV vaccination. Demographic characteristics, such as sex, age, alcohol consumption, smoking status, and region of residence in Romania, of the study cohort are shown in Table 1. Both written and verbal consent were obtained for the patient and control groups following the Declaration of Helsinki. This study was approved by the Ethics Council of the Fundeni Clinical Institute, Bucharest, Romania (no. 28640/25.05.2022).

The following eligibility criteria were required: inclusion criteria—patients in the CHB group were identified as individuals diagnosed with positive HBs antigen (HBsAg), negative anti-HBs antibodies, and negative anti-HBcore antibody. Their HBV DNA levels were assessed during two follow-up visits within one year, with an interval of at least six months between each visit. For liver cirrhosis among HBV-infected patients, laboratory tests were confirmed by liver biopsy and sonography or computed tomography.

For liver cirrhosis in HBV-infected patients, laboratory tests such as HBsAg, HBV DNA level, ALT, AST, total bilirubin, hemoglobin, and platelet count were performed, and for these patients, liver cirrhosis was confirmed by liver biopsy and sonography or computed tomography.

All participants were Caucasian and over 18 years of age. Participants were excluded if they were younger than 18 years or had evidence of co-infection with human immunodeficiency virus (HIV), hepatitis C virus (HCV), hepatitis D virus (HDV), autoimmune hepatitis, alcoholism-related cirrhosis, and hepatocellular carcinoma (HCC).

### 2.2. Biochemical and Viral Marker Analysis

Clinical biochemistry markers, including classical biomarkers such as alanine aminotransferase (ALT), gamma-glutamyl transpeptidase (GGT), aspartate aminotransferase (AST), alkaline phosphatase (ALP), and total and direct bilirubin, were conducted using an enzymatic method. This method employed the Dimension^®^ EXL™ 200 analyzer (Siemens Healthcare GmbH, Germany, Forchheim) in conjunction with specific detection kits. Virological markers, such as hepatitis B surface antigen (HBsAg), hepatitis B surface antibody (anti-HBs), hepatitis B e antigen (HBeAg), hepatitis B e antibody (anti-HBe), hepatitis B core antibody (anti-HBc), and α-fetoprotein (AFP), were quantified on Abbott ARCHITECT (Abbott, Green Oaks, IL, USA) following the manufacturer’s recommended procedures, using chemiluminescent principles. Quantitative HBV DNA levels were assessed using real-time polymerase chain reaction (RT-PCR) with the Montania 4896 device and Bosphore HBV Quantification Kit (Anatolia Geneworks, Turkey, Istanbul) according to the manufacturer’s instructions.

### 2.3. DNA Extraction

To perform DNA extraction, each participant in the study donated 5 mL of blood, collected in EDTA or citrate tubes. The kit used for isolating the DNA was the QIAamp DNA Blood Mini^®^ kit provided by QIAGEN (Hilden, Germany). The kit contained tubes with a silica membrane that caught the DNA from 200 µL of whole blood. After extracting the DNA from the blood, we tested the concentration and purity of the DNA using a nanophotometer provided by Implen. The targeted concentration was above 20 ng/µL, while the ideal purity report was between 1.7 and 1.9. After extraction, we stored the DNA in low-binding tubes at −18 °C.

### 2.4. HLA Genotyping

Following Immucor (Mia Fora NGS Flex, Norcross, GA, USA) instructions, we performed the genotyping of 11 HLA genes: 3 genes belonging to class I (HLA-A, HLA-B, and HLA-C) and 7 HLA class II genes. The class II HLA genes we took into account were HLA-DQA1 and HLA-DQB1 from the DQ locus, HLA-DPA1 and HLA-DPB1 from the DP locus, and HLA-DRB1, HLA-DRB3, HLA-DRB4, and HLA-DRB5 from the DR locus. Prior to the sequencing, a long-range PCR was performed to increase the quantity of DNA. A 10 μL genomic DNA sample was combined with 15 μL of an all-in-one master mix containing gene-specific primers, dNTPs, PCR buffer, and enzymes in 96-well PCR plates. In the end, the total PCR reaction volume was 25 μL. The PCR was performed in a specific thermal cycler from Applied Biosystems (Veriti 96-Well Thermal Cycler, Foster, CA, USA), one of the few compatible with the sequencing method. To obtain an optimal DNA concentration, the program used on the thermal cycler consisted of 10 s of initial hold at 98 °C, followed by denaturation at 98 °C, annealing at a temperature of 60 °C for 15 s, and a final extension performed at 66 °C for 10 min for a total of 25 cycles. After this first round of amplification, the PCR products were cleaved into smaller fragments using an enzymatic mix; afterward, they were end-repaired, and in the end, they were A-tailed and barcoded. After each fragment was given its specific identity using these barcodes, the DNA with 500 up to 900 bp was selected using the Pippin Prep system (Sage Science, Inc., Beverly, MA, USA). The eluate was amplified with Illumina primers necessary for cluster generation. The amplified sequencing library was cleaned, quantified, and prepared for Illumina sequencing. The data generated by the Illumina MiniSeq Sequencer (Illumina, San Diego, CA, USA) were processed afterward with the MIA FORA FLEX version 5.2 alignment software.

### 2.5. Statistical Analysis

We investigated the associations between HLA diversity and the risk of HBV infection, which were divided into major categories (control group, CHB group, and LC group). Individuals carrying two different alleles for each HLA locus were defined as heterozygotes. Homozygosity was defined as the presence of 2 identical alleles for a locus. In this study, statistical analysis was conducted using MiDAS HLA [16] tool version 1.10.0, an R package integrated with the Bioconductor software version 3.18. The impact of HLA heterozygosity and HLA evolutionary divergence (HED) on hepatitis B risk was assessed with a logistic regression model, calculating odds ratios (ORs) and 95% confidence intervals (95% CI). A *p*-value of ≤0.05 was considered statistically significant. We applied the Benjamini and Hochberg (BH) correction method to the *p*-values [17].

## 3. Results

We tested the heterozygote advantage hypothesis in an HBV major group (CHB group and LC group), compared with a healthy group (Table 2).

Our study revealed a significant association between increased HLA class II heterozygosity and a decreased risk of disease. The heterozygosity of HLA-DQB1 (OR = 0.49, 95% CI = 0.31–0.76, *p* = 0.01277) and HLA-DRB1 (OR = 0.42, 95% CI = 0.24–0.77, *p* = 0.01855) were significantly associated with a lower risk of HBV infection.

To explore the HLA diversity effect on the hepatitis B virus infection risk, we performed a subgroup analysis.

To evaluate the evolutionary divergence of alleles, we used the method described in the study by Pierini et al. to calculate HED (HLA evolutionary divergence). HED was calculated for heterozygous individuals carrying two different alleles at the same HLA locus, according to the magnitude of sequence divergence between alleles [18].

Eight human MHC genes, HLA class I (HLA-A, HLA-B, and HLA-C) and class II (HLA-DRB1, HLA-DQA1, HLA-DQB1, HLA-DPA1, and HLA-DPB1), were analyzed in this study. We did not consider the loci HLA-DRB3, HLA-DRB4, and HLA-DRB5 because they were in linkage disequilibrium and expressed in linkage with HLA-DRB1 genes. Alleles at each locus were defined at a three-field resolution. These criteria resulted in the analysis of 37 alleles for HLA-A, 51 for HLA-B, 36 for HLA-C, 37 for HLA-DRB1, 19 for HLA-DQA1, 21 for HLA-DQB1, 10 for HLA-DPA1, and 27 for HLA-DPB1 for the CHB group and 36 alleles for HLA-A, 62 for HLA-B, 35 for HLA-C, 37 for HLA-DRB1, 21 for HLA-DQA1, 17 for HLA-DQB1, 9 for HLA-DPA1, and 27 for HLA-DPB1 for the healthy group.

**Figure 1:**
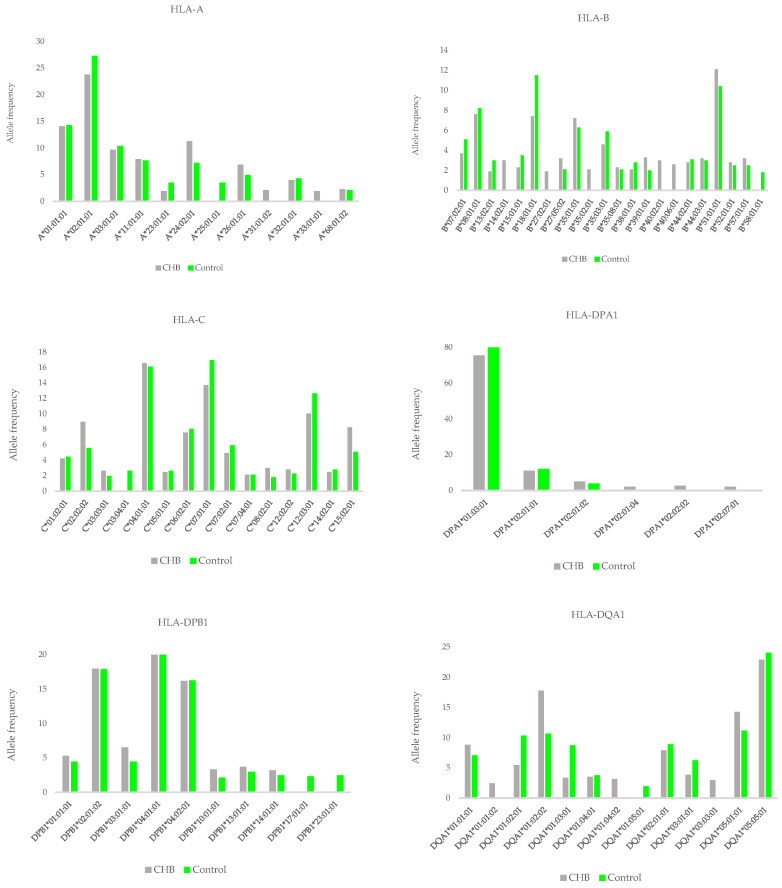

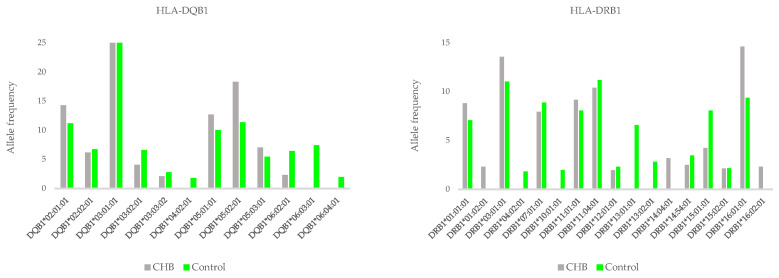
Frequency distribution of HLA alleles in the CHB group and the control group.

Figure 1 shows the frequencies of the most important HLA class I and class II alleles. The number of alleles varied among the different HLA loci, reflecting general differences in allelic diversity among the loci.

HLA-DPA1 and HLA-DPB1 were much less polymorphic than the other loci, particularly compared to a number of frequent alleles. Alleles of class I HLA-A and HLA-C and alleles of class II DQA1 and DQB1 carried a very similar amount of information that was lower than in HLA-B, which was by far the most polymorphic of the eight loci.

We focused on the most common alleles (allele frequency >5%).

For HLA-A, only four alleles showed a frequency >5%. These alleles were A*02:01:01 (27.3%), A*01:01:01 (14.3%), A*03:01:01 (10.4%), and A*11:01:01 (7.7%).

For HLA-B, six alleles showed a frequency >5%. These alleles were B*18:01:01 (11.5%), B*51:01:01 (10.4%), B*08:01:01 (8.2%), B*35:01:01 (6.3%), B*35:03:01 (5.9%), and B*07:02:01 (5.10).

For HLA-C, six alleles showed a frequency >5%. These alleles were C*07:01:01 (16.94%), C*04:01:01 (16.12%), C*12:03:01 (12.6%), C*06:02:01 (8.6%), C*07:02:01 (5.92%), C*02:02:02 (5.59%), and C*15:02:01 (5.10%).

For HLA-DPA1, only two alleles showed a frequency >5%. These alleles were DPA1*01:03:01 (79.93%) and DPA1*02:01:01 (12.01%).

For HLA-DPB1, three alleles showed a frequency >5%. These alleles were DPB1*04:01:01 (36.18%), DPB1*02:01:02 (17.93%), and DPB1*04:02:01 (16.28%).

For HLA-DQA1, six alleles showed a frequency >5%. These alleles were DQA1*05:05:01 (24.01%), DQA1*01:02:02 (10.69), DQA1*01:02:01 (10.36%), DQA1*02:01:01 (8.8%), DQA1*01:03:01 (8.72%), and DQA1*01:01:01 (7.07%).

For HLA-DQB1, four alleles showed a frequency >5%. These alleles were DQB1*03:01:01 (25.16%), DQB1*05:02:01 (11.35%), DQB1*02:01:01 (11.18%), and DQB1*05:01:01 (10.03%).

For HLA-DRB1, six alleles showed a frequency >5%. These alleles were DRB1*03:01:01 (11.18%), DRB1*03:01:01 (11.02%), DRB1*16:01:01 (9.38%), DRB1*07:01:01 (8.88%), DRB1*11:01:01 (8.06%), and DRB1*01:01:01 (7.07%).

Among all analyzed HLA alleles of class I and class II, HLA-B (OR = 0.87, 95% CI = 0.80–0.96, *p* = 0.07545) showed the highest divergence but was not statistically significant.

The high degree of polymorphism of HLA-I genes led to substantial diversity in peptide antigen presentation to T cells. This polymorphism also resulted in variations in the intracellular folding and assembly of the proteins, as well as in their interactions with immune receptors at the cell surface. The HLA-I genotype with two alleles that had more divergent sequences presented a more diverse array of immunopeptidomes. The highly polymorphic genes of the HLA genes play a key role in adaptive immunity.

## 4. Discussion

The impacts of hepatitis B virus genotypes on clinical outcomes, chronicity, disease progression, risk of hepatocellular carcinoma, promoter mutations, and response to interferon therapy are well recognized. Based on genomic sequences or the gene coding for the surface antigen, HBV is classified into 10 genotypes from A to J, which are defined as >7.5% genetic divergence within the full genome sequence of the virus The prevalence of HBV genotypes varies worldwide, with genotypes A and D being the most common in Europe. In Romania, Constantinescu et al. showed that the most prevalent genotype in patients with chronic HBV infection is D (60.5%), followed by genotype A (8.1%). Genotype D is the most likely genotype to be associated with basal core promoter (BCP) A1762T/G1764A and pre-core (PC) G1896A mutations [19].

The HLA system represents a set of molecules on the cell surface that are key for the immune system, and these molecules are highly polymorphic. Due to the variability of the amino acid sequence, different HLA molecules bind differently to peptides. Therefore, certain HLA molecules are more efficient at binding and presenting certain peptides than others. This diversity enables the immune system to respond to an immense category of pathogens [20].

HLA class I and II genes are highly polymorphic and encode several glycoproteins found on the cell membrane [21]. Thanks to advances in analyzing technologies such as NGS, the analysis of the HLA genes provides valuable information on the molecular structures and genetic mechanisms of various diseases.

The allelic diversity of HLA genes has a significant impact on immunity, influencing how the body responds to infections and cancers. This diversity plays an essential role in the selection of T cells and in determining the efficiency of the immune response.

In evolutionary biology, the hypothesis that HLA heterozygotes possess greater resistance to infectious diseases is a key explanation for the extraordinary diversity of HLA genes and the persistence of this diversity throughout evolution [22]. The heterozygote advantage hypothesis for HLA genes proposes that HLA heterozygosity in individuals offers an immunological advantage over those who are homozygous. This is due to the increased ability of the immune system to react to a greater variety of antigens [12,23]. According to this hypothesis, HLA class I heterozygous genotypes present a wider variety of tumor antigens to lymphocytes [24]. The heterozygous advantage hypothesis was expanded to also define the divergent allele advantage when assessing the sequence level. In short, HLA alleles diversity at the same locus can improve the functional ability to present peptide antigens, thereby enhancing protection against pathogens and tumors by the more effective activation of T-cell immunity [25]. More than that, the ratio of mutations changing the structure may be important for demonstrating that more divergent alleles are more “fit” than the ones with similar alleles [26].

Nonsynonymous mutations that alter a protein’s amino acid sequence are typically neutral for most genes, but for HLA genes, the range of peptides that the HLA protein may load and transmit to the T cell receptor is determined by amino acid changes in the domain that recognizes antigens [27]. In our study, we explored the importance of HLA heterozygosity and the protective effect in HBV infection. HLA diversity (HED) has been correlated with susceptibility to numerous diseases. Previous studies have suggested that homozygous individuals for HLA alleles face a significant disadvantage, due to their more limited peptide repertoire than heterozygous individuals. A high HED among an individual’s HLA alleles may facilitate the expression of a more varied immunopeptidome. This diversity is crucial for presenting tumor-associated antigens, necessary for the efficacy of graft-versus-leukemia-mediated anti-tumor responses and an effective anti-cancer immune response [28]. Current research has demonstrated that heterozygosity at HLA II is associated with favorable clinical outcomes, especially in viral infection. According to this premise, research into hepatitis C virus infection has identified specific HLA class II molecules that confer protection against the severity of liver complications caused by the hepatitis C virus infection [29]. Additionally, a heterozygote advantage in the context of hepatitis C virus (HCV) infection has been described by Hraber et al. According to their findings, the subjects with HCV infection had much lower percentages of HLA-DRB1 heterozygosity than those without infection. HLA-DRB1 genotype, together with genetic and epigenetic factors, could be an important tool for understanding treatment options [30]. Studies have shown that individuals with diverse HLA class II genes (heterozygosity) are less likely to develop chronic hepatitis B or HBV-related liver cancer [31].

Similar findings have been reported, where the genetic diversity of HLA genes was associated with lower susceptibility to human immunodeficiency virus (HIV) infection [32,33].

Other findings in recent studies suggest that in certain viral infections, the heterozygosity of HLA is connected with favorable disease outcomes. Supporting the concept of heterozygote advantage in HLA II, research has revealed that individuals who are homozygous for HLA-DRB1*16:02 and HLA-DRB1*03:01 have a reduced capacity to produce effective antibodies against viruses like human adenovirus (HAdV)-C and human herpesvirus (HHV) species, compared to heterozygous individuals [34].

Data indicate that heterozygosity at HLA class I loci confers an immunological advantage in human T cell lymphotropic virus type I (HTLV-I) infection. Thus, heterozygous individuals exhibit lower viral loads, which can be attributed to a better cytotoxic T cell-mediated immune response. This observation underscores the importance of genetic diversity in determining susceptibility to infection and disease outcome [35].

Significant differences were observed between Crohn’s disease patients and ulcerative colitis patients, with different HLA II variants playing an important role and with a heterozygous advantage noted in ulcerative colitis. These findings suggest that the adaptive immune response to the colonic environment is a key factor in the pathogenesis of inflammatory bowel diseases [15]. Studies exploring the relationship between HLA and autoimmune diseases have indicated that increased HLA heterozygosity is associated with a more robust immune response and favorable disease progression [36].

The association between HLA heterozygosity and reduced cancer risk was first established in non-Hodgkin’s lymphoma (NHL), where distinct patterns of HLA homozygosity were linked to different NHL subtypes. Wang et al. extended this research by suggesting a broader role for HLA diversity in cancer prevention, positing that homozygous HLA genotypes increase vulnerability to NHL [37].

Analysis of the UK Biobank dataset demonstrated that increased HLA class II diversity is statistically associated with a lower incidence across a broad spectrum of cancer types [38]. The immunosurveillance hypothesis posits that HLA genetic diversity enhances the immune system’s capacity to recognize and eliminate tumor-associated antigens. Heterozygous HLA genotypes are predicted to facilitate antigen presentation, thereby amplifying cytotoxic T lymphocyte responses and potentially reducing the incidence of solid organ tumors. A rigorous analysis of a substantial dataset comprising 29,343 European individuals diagnosed with 12 different cancer types failed to support the immunosurveillance hypothesis. The study’s inability to identify a link between HLA homozygosity and overall cancer incidence challenges the prevailing paradigm that HLA heterozygosity confers a protective effect against cancer development [39].

Several limitations apply to the present study. The participants in our study were primarily Caucasian. The role of HLA diversity in hepatitis B susceptibility requires further investigation in populations with different genetic backgrounds. Due to the relatively small patient number, the results of the current work should be expanded to a larger cohort in order to further validate the results. Overall, more patients are needed to provide more definitive evidence regarding the association between HLA heterozygosity and hepatitis B virus infection risk.

## 5. Conclusions

The heterozygous advantage of the HLA class II genes in the context of hepatitis B has been studied to understand how genetic diversity can influence the response to this infection and the favorable outcome of HBV infection. In conclusion, the present data suggest a potential association between HLA diversity and the risk of HBV infection. Notably, HLA class II DQB1 heterozygosity was associated with a protective effect against HBV infection in a Romanian population. These findings underscore the importance of genetic factors in the susceptibility and resistance to HBV, highlighting the need for further research to understand the mechanisms underlying this association.

## Figures and Tables

**Table 1:** Demographic characteristics of the study population.

Variables	CHB Group	LC Group	Control Group	*p*-Value	*p*-Value	*p*-Value
	Number (%)	Number (%)	Number (%)	CHB vs. LC	CHB vs. Control	LC vs. Control
	(n = 284)	(n = 43)	(n = 304)			
Gender						
Male	140 (49.30)	20 (46.51)	186 (61.18)	0.734	0.004	0.067
Female	144 (50.70)	23 (53.49)	118 (38.82)			
Age (years)						
<40	110 (38.73)	10 (23.26)	234 (76.97)	0.071	<0.001	<0.001
40–49	42 (14.79)	5 (11.63)	63 (20.72)			
50–59	55 (19.37)	15 (34.88)	7 (2.30)			
≥60	77 (27.11)	13 (30.23)	0 (0.00)			
Region of residence						
Muntenia	183 (64.44)	26 (60.47)	151 (49.67)	<0.001	<0.001	<0.001
Oltenia	84 (29.58)	4 (9.30)	116 (38.16)			
Moldavia	11 (3.87)	7 (16.28)	4 (1.32)			
Dobrogea	3 (1.06)	3 (6.98)	0 (0.00)			
Crisana	1 (0.35)	1 (2.33)	0 (0.00)			
Transylvania	2 (0.70)	2 (4.65)	33 (10.86)			
Area of residence						
Urban	135 (38.03)	26 (60.47)	162 (53.29)	0.005	<0.001	0.377
Rural	149 (61.97)	17 (39.53)	142 (46.71)			
Alcohol consumption						
Yes	14 (4.93)	4 (9.30)	9 (2.96)	0.241	0.218	0.040
No	270 (95.07)	39 (90.70)	295 (97.04)			
Cigarette smoking						
Yes	39 (13.73)	6 (13.95)	18 (5.92)	0.969	0.001	0.052
No	245 (86.27)	37 (86.05)	286 (94.08)			

CHB—chronic hepatitis B; LC—liver cirrhosis; *p* < 0.05—statistical significance.

**Table 2:** Associations between HLA heterozygosity at the class II locus and the risk of HBV infection.

HLA Status	Estimate OR, (95% CI)	Adjusted *p*-Value (BH) with Significance	Total	Total Percentage	Control Group	Control Group Percentage	HBV Group	HBV Group Percentage
DQB1 heterozygote	0.49 (0.31–0.76)	0.01277	538	83.93%	270	88.82%	268	79.53%
DRB1 heterozygote	0.42 (0.24–0.77)	0.01855	583	90.95%	287	94.41%	296	87.83%

OR—Odds ratio; 95% CI—95% confidence interval; BH—Benjamini and Hochberg correction; *p* < 0.05—statistical significance; HBV—hepatitis B virus. OR and 95% CI were calculated using logistic regression models. The number of heterozygotes at the HLA locus was taken as a continuous variable in the logistic regression models.

## Data Availability

Data available on request from the authors due to restrictions, e.g. privacy or ethical.
